# Surface electromyographic activity of the erector spinae and multifidus during arm- and leg-ergometer exercises in young healthy men

**DOI:** 10.3389/fphys.2022.974632

**Published:** 2022-11-25

**Authors:** Daichi Shima, Yukihide Nishimura, Takamasa Hashizaki, Yuta Minoshima, Tatsuya Yoshikawa, Yasunori Umemoto, Tokio Kinoshita, Ken Kouda, Fumihiro Tajima, Yoshi-Ichiro Kamijo

**Affiliations:** ^1^ Department of Rehabilitation Medicine, Wakayama Medical University, Wakayama, Japan; ^2^ Division of Rehabilitation Medicine, Wakayama Medical University Hospital, Wakayama, Japan; ^3^ Department of Rehabilitation Medicine, Iwate Medical University, Morioka, Japan; ^4^ Department of Rehabilitation Medicine, Dokkyo Medical University Saitama Medical Center, Saitama, Japan

**Keywords:** paraspinal muscles, exercise therapy, rehabilitation medicine, arm-crank exercise, cycling exercise

## Abstract

**Objectives:** Ergometer exercise was considered a new loading method that can be used for participants who are unable to assume the core strengthening exercise posture commonly used to strengthen the erector spinae and multifidus. This study aimed to investigate with healthy participants whether arm and leg ergometers could be used for core strengthening exercises and whether different exercise sites would affect the results.

**Methods:** The study was conducted with 15 healthy adult male participants aged 20–35 years. The intervention consisted of arm- and leg-ergometer exercises performed by the participants. The exercise protocol consisted of three 1-min sessions (rest, 50W, and 100 W), which were measured consecutively. Surface electromyography (sEMG) was measured during the sessions. Maximal voluntary contraction (MVC) of the erector spinae and multifidus was also measured, during which sEMG was measured. The sEMG during ergometer exercise was calculated as a percentage of the MVC (calculated as % MVC). The root mean square (RMS) was recorded from the sEMG activity. Muscle activity of the erector spinae and multifidus was compared between ergometer exercises and between intensity levels. Heart rate (HR) was recorded by electrocardiogram.

**Results:** In the arm-ergometer exercise, the % MVC values of the erector spinae were 6.3 ± 3.1, 10.9 ± 5.4, and 16.9 ± 8.3% at rest, 50 W, and 100 W conditions, respectively. The multifidus was 4.6 ± 2.9, 9.2 ± 5.6, and 12.6 ± 7.6% at rest, 50 W, and 100 W conditions, respectively. The respective % MVC values during the leg-ergometer exercise were 3.8 ± 1.7, 7.2 ± 3.8, and 10.4 ± 4.0% at rest, 50 W, and 100 W conditions, respectively. Leg-ergometer exercises were 2.6 ± 2.1, 6.9 ± 5.7, and 10.3 ± 6.8% at rest, 50 W, and 100 W conditions, respectively. The activities of the two muscles increased at comparable levels with increased workload in both types of exercises (*p* < 0.01, each). HR increased with the increased workload and the increase was larger during arm-than leg-ergometer exercises.

**Conclusion:** These results demonstrate that both arm- and leg-ergometer exercises are potentially alternative methods for erector spinae and multifidus training for healthy participants. Further research is needed to target elderly.

## 1 Introduction

The erector spinae and multifidus, i.e., paraspinal muscles, have a cephalad attachment at the medial portion of the transverse and accessory processes of the spine and a caudal attachment at the iliac crest. Some fibers from the fifth lumbar vertebra longest muscle stop at the sacrum (Bogduk, 1980; Bustami, 1986; MacIntosh and Bogduk, 1987). The lumbar multifidus muscle originates at the spinous processes of the first to the fifth lumbar vertebra and spans vertebrae 2 to 5. Most fibers attach cephalad at the mastoid process, with the deeper fibers attaching caudally at the sacrum, soft tissue covering the sacrum, and erector spina tendon membrane (MacIntosh and Bogduk, 1987; Rosatelli et al., 2008). The paraspinal muscles work to control the movement of the entire spine and to ensure segmental stability (Bergmark, 1989), which is essential for movement stability. Recent studies indicate that spinal degeneration is a progressive process with specific gear-up periods in human life (Ekşi et al., 2022) and that aging and lumbar degeneration increase fatty infiltration of the paraspinal muscles and decrease muscle mass (Sasaki et al., 2017; Özcan-Ekşi et al., 2019). Fatty infiltration decreases the quality of the paraspinal muscles, as adipose tissue that develops within the muscle is not contractile (MacDonald et al., 2009; MacDonald et al., 2010; Macdonald et al., 2011). Patients with fatty infiltrated multifidus at the L4-L5 level are four times more likely to have more intense low back pain (Özcan-Ekşi et al., 2021) Further, weakness of the back extensors is associated with decreased risk of falls, spinal column mobility, and quality of life (Miyakoshi et al., 2005; Kasukawa et al., 2010). A large muscle cross-sectional area of the multifidus is important for maintaining sagittal alignment of the spine in the elderly (Banno et al., 2017). Indeed, the multifidus accounts for 20% of the extension moment at the L4-L5 level, while the erector spinae account for 30% (Bogduk et al., 1992). Furthermore, in addition to the lumbar multifidus, the transversus abdominis, diaphragm, and pelvic floor muscles co-contract to provide stability (Hodges et al., 1997; Sapsford et al., 2001; Sapsford and Hodges, 2001; Moseley et al., 2002). Since previous evidence indicates that regular training increases the cross-sectional area of the paraspinal muscles (Storheim et al., 2003), preventing and strengthening paraspinal muscle atrophy may reduce the risk of falls and improve quality of life and postural stability.

Commonly performed exercises for the trunk muscles use core stabilization and strengthening exercises. Core stabilization exercises are designed to promote simultaneous activation of the transversus abdominis and multifidus. These deep stabilizing muscles attach to the thoracolumbar fascia and provide stability to the spine by increasing intra-abdominal pressure (Vleeming et al., 2014). The method begins with the abdominal drawing-in maneuver in bed, followed by abdominal retraction exercises while sitting, crawling on all fours, and standing, depending on the proficiency level (Hlaing et al., 2021). In contrast, strengthening exercises are designed to increase overall trunk muscle strength, and control and improve general spinal stability. The method begins with exercises on a bed, as in stabilization exercises, followed by side bridges and arm and leg exercises in the crawling position (Hlaing et al., 2021). However, frail, elderly patients have trouble exercising while standing, due to the risk of falling. In clinical settings, these individuals have difficulty in supine and prone positions in bed when in a round-back posture. Additionally, if there is a decline in cognitive function, the patient may not be able to perform more complex tasks. It is necessary to construct a loading method that can be performed safely and easily even by such patients.

Ergometer exercise is known as cardiopulmonary training, including improving cardiovascular function and ventilation threshold (Lovell et al., 2010; Vogel et al., 2011; Lovell et al., 2012). Compared to treadmill walking or stair climbing, the ergometer, a seated exercise, is safer with no risk of falling. Additionally, ergometer exercise has been performed with patients with various cardiac conditions (Rupprecht et al., 2017; Atef and Abdeen, 2021) and is a safe loading method for elderly patients with a history of cardiac disease. Some studies using sEMG on paraspinal muscles during ergometer exercise have reported that paraspinal muscle activity was obtained during both arm and leg movements (Marks et al., 2012; Xiao et al., 2014). With each increase in load during arm-ergometer exercise, the paraspinal muscle activity also increased (Marks et al., 2012). In other studies, while the exercise loading method varied, paraspinal muscle activity during biceps curl (Oliveira Ade and Gonçalves, 2009) and squat exercises (Clark et al., 2019) also increased due to the increased loading. However, whether paraspinal muscle activity increases in a load-dependent manner during leg-ergometer exercise has remained unclear. Furthermore, the extent by which ergometer exercise activates paraspinal muscle activity compared to previously reported percentages of MVC (% MVC) during trunk training is unknown. This study aimed to investigate whether arm and leg ergometers can be used for trunk strengthening exercises and whether differences in the exercise site affect the results. Therefore, sEMG was recorded in young, healthy participants and electromyographic activities of erector spinae and multifidus were compared between arm- and leg-ergometer exercises and at similar loads.

## 2 Methods

### 2.1 Participants

Fifteen healthy male participants aged 20–35 years were recruited for this study. Their physical characteristics are summarized in Table 1. The study protocol and consent forms were approved by the Human Ethics Committee of Wakayama Medical University (approval #2609). The study was conducted in accordance with the Declaration of Helsinki. All individuals were informed about the purpose and risks of the study before the consent. At the same time, their medical history was obtained through an interview. Exclusion criteria were previous spine, hip, or knee procedures or surgery; had low back pain history; pain in the arm or leg; congenital anomalies of the spine or lower extremities; cardiovascular disease; pulmonary disease; diabetes and other metabolic diseases; muscular disease; and known spinal disease. Screening of pathology and spinal disease after surgery were assessed by interview. None of the exclusion criteria were met. One person was excluded due to a mechanical issue with the equipment on the day of the test (final sample: 14).

**TABLE 1:** Anthropometric characteristics of the study participants (n = 14).

	
Age (years)	27.1 (3.9)
Height (cm)	171.2 (5.1)
Weight (kg)	62.4 (6.9)
BMI (kg/m^2^)	21.3 (2.4)

Values are means (SD). BMI, body mass index.

### 2.2 Measurements

#### 2.2.1 Arm- and leg-ergometer exercises

**FIGURE 1:**
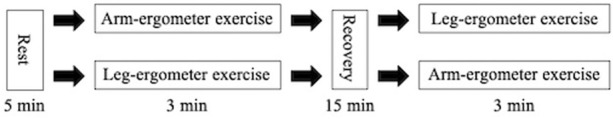
The diagram of experimental design.

**FIGURE 2:**
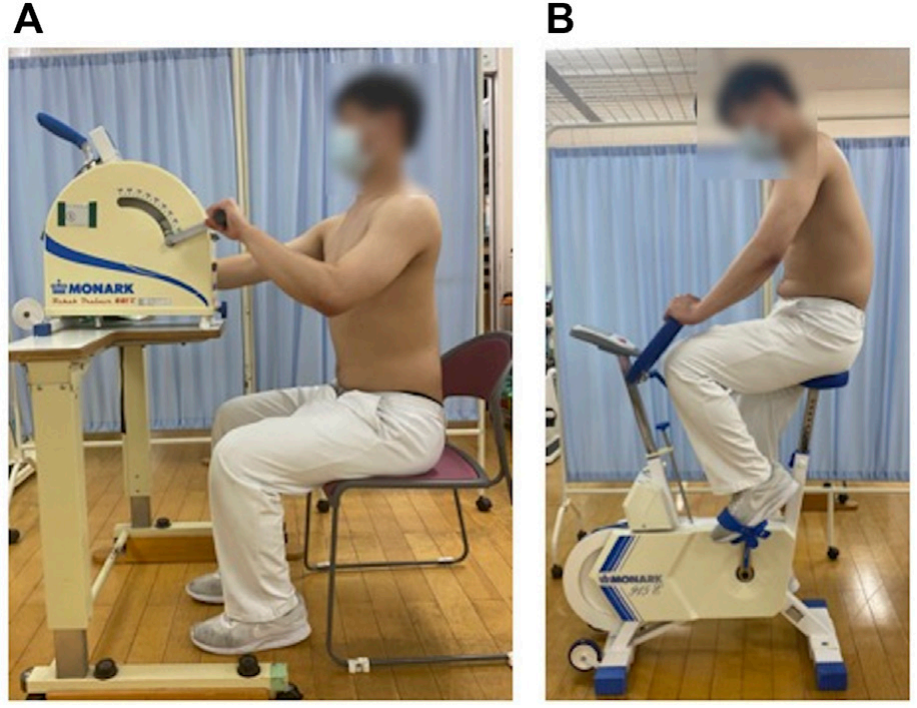
Arm- and leg-ergometer exercises **(A)** Starting posture for arm-ergometer exercise **(B)** Starting posture for leg-ergometer exercise.

A diagram of experimental design of the ergometer exercise conducted in this study is shown below (Figure 1). For the arm-ergometer exercise, the participant was seated in a chair and asked to hold the device’s handle. The height of the ergometer was adjusted so that the right shoulder joint was at 90° flexion and the right elbow joint at 0° extension. In the starting position, the participant was instructed to sit at the edge of the chair so that his back did not touch the backrest while electrodes were applied to his back (Figure 2A). The measurements were divided into three sessions. In the first session, the participant was allowed to rest for 1 min in the starting posture. In the second session, the participant performed a 1-min ergometer exercise with a 50 W load. The third session also had the participant perform a 1-min ergometer exercise with a 100 W load. These sessions were held successively. One set of each of the above three sessions was performed on the arm and leg ergometer. The rest between sets was approximately 15 min. For the leg ergometer, the participant sat on the saddle with his foot on the ergometer pedal. The saddle height was adjusted so that the right hip joint was at 0° extension and right knee joint was at 0° extension. Since there was no backrest, a relaxed posture was assumed, which was used as the starting position (Figure 2B). The leg-ergometer exercise also consisted of three sessions, with the same content as the arm-ergometer exercise. Exercise duration was 2 min, excluding rest, in both ergometer exercises.

#### 2.2.2 Isometric maximal voluntary contraction

Measurement of back muscle MVC was performed with reference to previous studies (Tsuboi et al., 2018). The participant was in the prone position over two treatment tables that could be lowered. The arms and body rested over the table, while the anterior superior iliac spines were positioned on the edge of the other table, supporting the lower body. Three straps were used to stabilize the hips, knees, and ankles. MVCs were measured with the participant in the prone position, with the hands placed behind the head while the head was midline, and elbows positioned out to the sides. The resistance area to the back was applied to the subscapular angle at the middle of the back between the shoulder blades. During this time, EMG was continuously measured. Participants were given 5 s to reach their maximum effort. The test was performed three times with a 30-s rest interval and the maximum force was recorded. Three 5-s MVCs were recorded, and after obtaining the RMS for each second, the maximum value was used as the MVC value.

### 2.3 Experimental design

The study was conducted with a repeated measures design. All participants exercised on arm- (915E, Monark, Varberg, Sweden) and leg-ergometers (881E, Monark, Varberg, Sweden) on the same day at approximately 6:00 p.m. and their back muscle isometric MVCs were measured. The order of the arm- and leg-ergometer exercises was randomly assigned for each participant. Before the measurement, the protocol was explained to the participant, and a pair of silver-silver chloride cup electrodes for sEMG and another for the electrocardiogram (ECG; Bedside monitor, BSM-2401, Nihon Kohden, Tokyo) were placed on the muscle of interest and on the chest, respectively. The participant sat on either a chair for the arm ergometer or the saddle of the leg-ergometer. After confirming a stable heart rate (HR) on the ECG, the recording of the sEMG was initiated. After the first sessions, the participant rested for approximately 15 min until HR returned to the pre-exercise rate. Subsequently, the participant was invited to do the other type of ergometer exercise. The ergometer exercises took approximately 1 hour to complete, including preparation and implementation.

### 2.4 Electromyography signal recording and analysis

**FIGURE 3:**
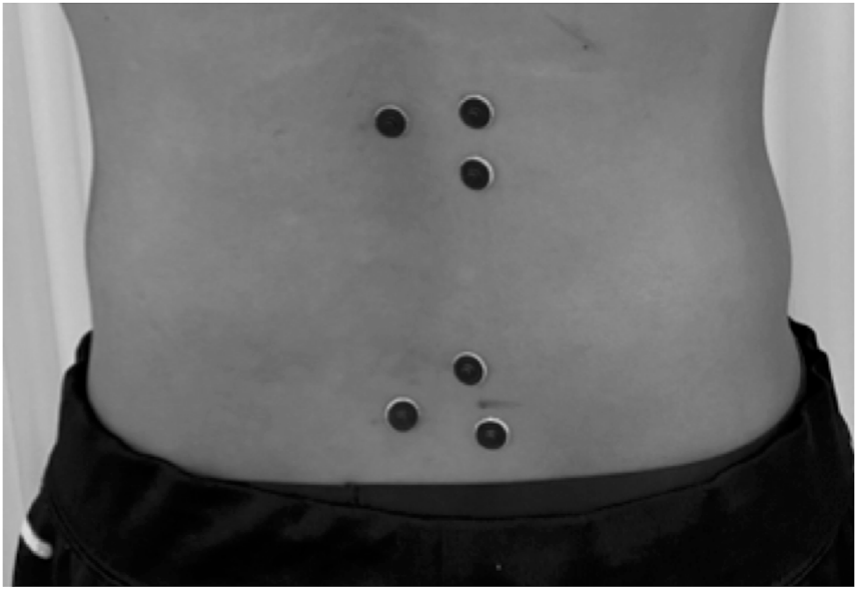
Electrode attachment sites. This is a back view, with the upper electrode showing the erector spinae and the lower electrode showing the multifidus.

The EMG activity was monitored during ergometer exercises and MVC. A pair of silver-silver chloride surface electrodes was placed on the right side of the erector spinae and multifidus after the respective skin areas were shaved and cleaned with an alcohol swab. The diameter of the recording electrode was 10 mm, and the two electrodes were 2 cm apart on each site. sEMG of the erector spinae and multifidus were measured in all participants. The electrode positioning on the selected muscles followed the protocol by the SENIAM project (Hermens et al., 2000), which is as follows: 1) the electrodes must be placed at two finger width lateral from the spinous process of first lumbar vertebra and 2) the multifidus electrodes must be placed on and aligned with a line from caudal tip posterior spina iliaca superior to the interspace between first lumbar spinous process and second lumbar spinous process interspace at the level of the fifth lumbar spinous process (Figure 3). The study was conducted using a cordless electromyography system (MQ air, Kissei Comtec, Matsumoto, Japan). The signals were bandpass filtered (8–500 Hz), digitized using an A/D converter (AIO-163202FX-USB, CONTEC, Osaka, Japan), then stored on a computer using recording software (Vital Recorder, Kissei Comtec, Matsumoto, Japan) at 1,000 Hz sampling rate.

RMS was obtained offline from stored sEMG signals using analysis software (BIMUTAS-Video, kissei Comtec, Matsumoto, Japan). To calculate RMS during the exercise on the ergometer, only the signal recorded between 20.4 and 43.2 s in each session was selected for each participant as during this period, increasing the load between sessions takes a few seconds. Additionally, the amount of load might not reach the setting during the initial period. The average value was calculated from the 20 points obtained by the above method. For each session, the RMS was averaged at rest, 50 W, and 100 W conditions in each participant. Changes (*Δ*) from the rest values were calculated at 50 W and 100 W conditions.

Similarly, for RMS during MVC, which was performed three times during the measurement, three points with elevated values were identified from the measurement data, and 5 seconds were picked up from each point with elevated values. The maximum value was then calculated from the 15 s of data obtained. The percentage of the RMS value obtained during the ergometer exercise was determined relative to the RMS value in the obtained MVC (denoted as % MVC).

### 2.5 Electrocardiogram signal recording and analysis

The ECG was monitored during ergometer exercise. The electrodes were placed on the anterior chest. HR was recorded at the end of each stage of the protocol, at rest and exercise at 50 W and 100 W conditions. Changes (*Δ*) from the rest values were calculated at 50 W and 100 W conditions.

### 2.6 Statistical analyses

One-way ANOVA for repeated measurements was used to analyze the % MVC of the erector spinae and multifidus and HR during arm- and leg-ergometer exercises. The Tukey-Kramer test was used to evaluate the differences among 50, 100 W, and rest conditions as post-hoc tests. In addition, a paired *t*-test was used for comparisons among the groups and *Δ* values. All values are expressed as mean (SD) unless otherwise stated. Statistical significance was set at *p* < 0.05. All data were analyzed using GraphPad Prism 7 (San Diego, CA). The sample size for this study was 15 participants, based on previous studies (Oliveira Ade and Gonçalves, 2009; Marks et al., 2012; Tsuboi et al., 2018). Statistical power was then calculated as a post-hoc test. The statistical power of this study was 70%.

## 3 Results

### 3.1 Percentage of maximal voluntary contraction of erector spinae during arm- and leg-ergometer exercises

**FIGURE 4:**
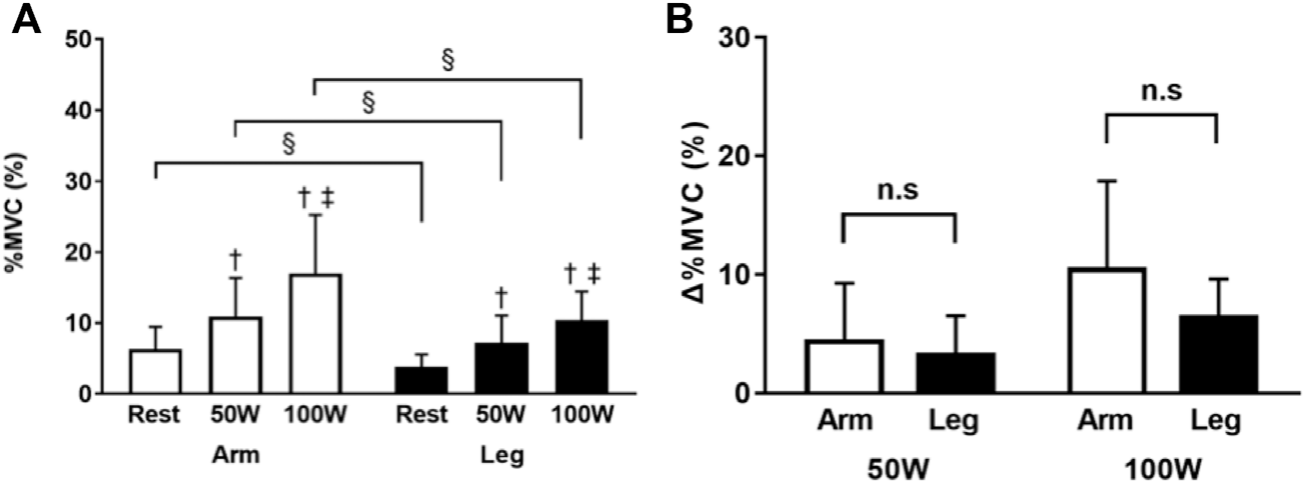
Percent of MVC of the erector spinae during arm- and leg-ergometer exercises **(A)** Values of percentage MVC (% MVC) at rest, 50W, and 100 W conditions of exercise **(B)** Changes (*Δ*) from the baseline (rest). Values are mean ± SD for 14 participants. †, ‡, compared with rest and 50 W conditions, respectively; §, different from that during the arm exercise at the level of *p* < 0.05; n. s, not significant.

In the arm-ergometer exercise, the % MVC values of the erector spinae were 6.3 ± 3.1, 10.9 ± 5.4, and 16.9 ± 8.3% at rest, 50 W, and 100 W conditions, respectively. The respective % MVC values during the leg-ergometer exercise were 3.8 ± 1.7, 7.2 ± 3.8, and 10.4 ± 4.0%. The % MVC values in both the arm- and leg-ergometers increased with increased workloads (50 W and 100 W conditions vs. rest condition, *p* < 0.01; 100 W condition vs. 50 W condition, *p* < 0.01, each). The differences in % MVC values were significantly higher for arm-ergometer exercise than for leg-ergometer exercise at rest, 50 W, and 100 W conditions (*p* < 0.05). However, there was no significant difference in the range of increase in % MVC, between arm- and leg-ergometer exercises (Figure 4).

### 3.2 Percentage of MVC of the multifidus during arm- and leg-ergometer exercises

**FIGURE 5:**
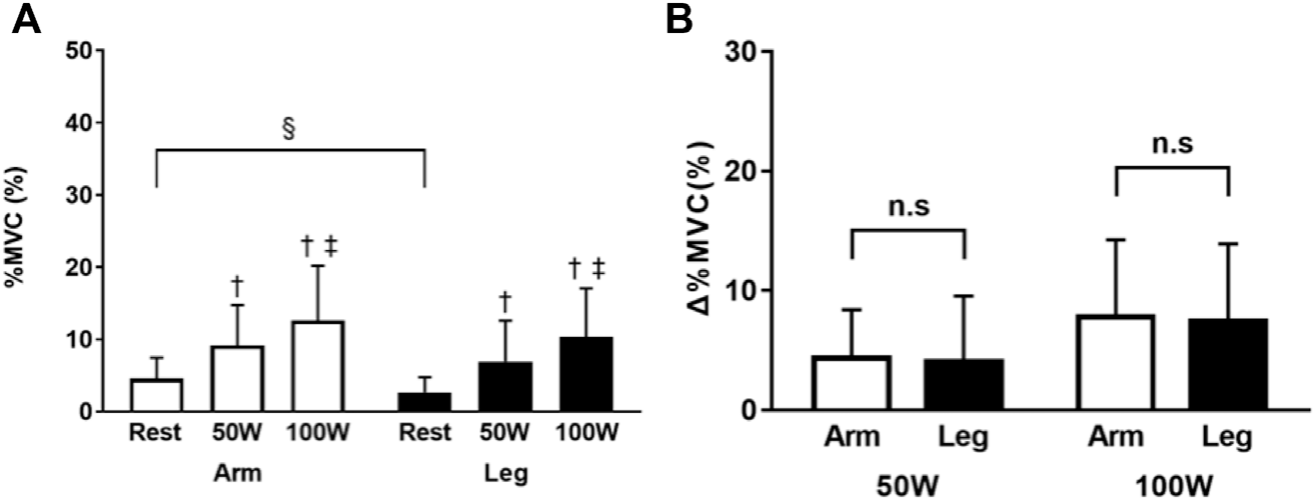
Percent of MVC of the multifidus during arm- and leg-ergometer exercises **(A)** Values of percentage MVC (% MVC) at rest, 50 W, and 100 W conditions of exercise **(B)** Changes (*Δ*) from the baseline (rest). Values are mean ± SD for 14 participants. †, ‡, compared with rest and 50 W conditions, respectively; §, different from that during the arm exercise at the level of *p* < 0.05; n. s, not significant.

In the arm-ergometer exercise, the % MVC values of the multifidus were 4.6 ± 2.9, 9.2 ± 5.6, and 12.6 ± 7.6% at rest, 50 W, and 100 W conditions, respectively. The respective % MVC values during the leg-ergometer exercise were 2.6 ± 2.1, 6.9 ± 5.7, and 10.3 ± 6.8%. The % MVC values increased with increased workloads (50 W and 100 W conditions vs. rest condition, *p* < 0.01; 100 W condition vs. 50 W condition, *p* < 0.01, all). The difference in % MVC values was significantly higher for arm-ergometer exercise than for leg-ergometer exercise at rest condition (*p* < 0.05). However, there was no significant difference between the two types of exercises at 50 W or 100 W conditions. There was also no significant difference in the range of increase in muscle activity of the multifidus between the arm- or leg-ergometer exercises (Figure 5).

### 3.3 Heart rate during arm- and leg-ergometer exercises

**FIGURE 6:**
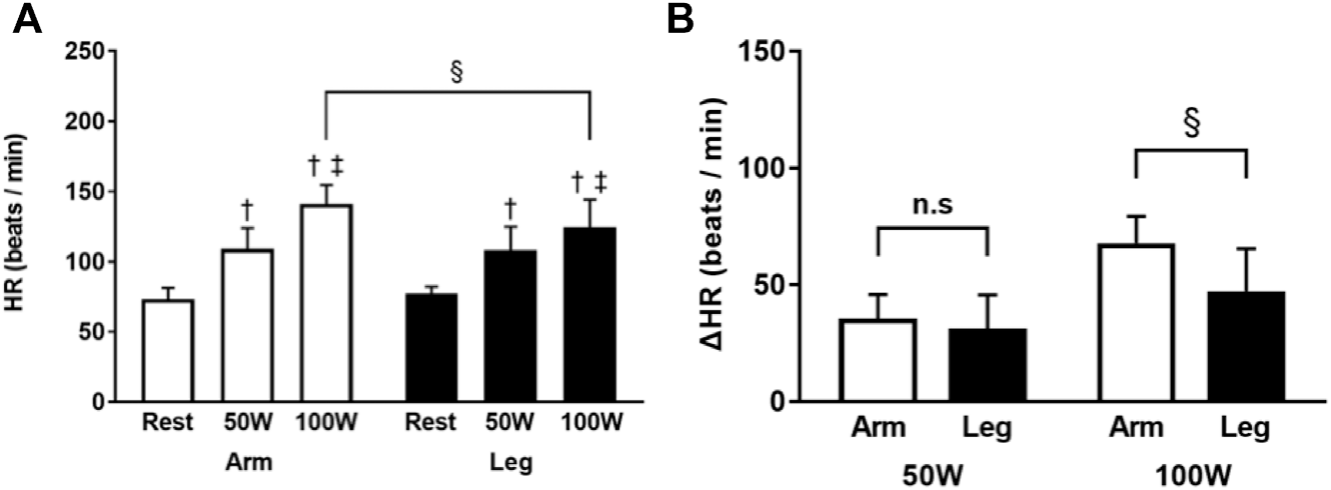
Heart rate of the erector spinae during arm- and leg-ergometer exercises **(A)** Heart rate (HR) at rest, 50 W, and 100 W conditions of exercise **(B)** Changes (*Δ*) from the baseline (rest). Values are mean ± SD for 14 participants. †, ‡, compared with rest and 50 W conditions, respectively; §, different from that during the arm exercise at the level of *p* < 0.05.

In the arm-ergometer exercise, the HR values were 73 ± 8, 109 ± 14, and 141 ± 13 beats/min at the rest, 50 W, and 100 W conditions, respectively. The respective HR values during the leg-ergometer exercise were 77 ± 5, 108 ± 16, and 124 ± 19 beats/min. HR increased with increased workloads in both ergometer exercises (50 W and 100 W conditions vs. rest condition, *p* < 0.01; 100 W vs. 50 W, *p* < 0.01, all). The difference in HR values was significantly higher for the arm-ergometer exercise than for the leg-ergometer exercise at only the 100 W condition. The range of HR increase in arm- and leg-ergometer exercise was not significantly different at the 50 W condition. However, at the 100 W condition, the range of HR increase in arm-ergometer exercise was significantly higher than that of the leg-ergometer exercise (*p* < 0.01) (Figure 6).

## 4 Discussion

The results of the current study show that at 50 W and 100 W conditions, both arm- and leg-ergometer exercises increased paraspinal muscle activity with increasing loads. Furthermore, in the erector spinae, arm-ergometer exercise was shown to promote more muscle activity. This is the first report comparing the effects of arm- and leg-ergometer exercise on the paraspinal muscles.

The spinal erector spinae activities obtained in this study were 10.9 ± 5.4% at 50 W condition and 16.9 ± 8.3% at 100 W condition for the arm-ergometer exercise, and 7.2 ± 3.8% at 50 W condition and 10.4 ± 4.0% at 100 W condition for the leg-ergometer exercise. In the trunk strengthening exercises from the previous study, the back bridge had approximately 25% of the erector spinae MVC, the right side bridge had approximately 15% of the right erector spinae MVC and 10% of the left erector spinae MVC, and the exercise to raise the right arm and left leg in the crawling position had approximately 20% of the right erector spinae MVC and 15% of the left erector spinae MVC (Imai et al., 2010). The muscle activity of the multifidus obtained in this study was 9.2 ± 5.6% at 50 W condition and 12.6 ± 7.6% at 100 W condition in the arm-ergometer exercise. For leg-ergometer exercise, the muscle activity at 50 W and 100 W conditions was 6.9 ± 5.7% and 10.3 ± 6.8%, respectively. For the multifidus, the results of the trunk strengthening exercises in a previous study reported approximately 40% for the back bridge, 20% for the right side bridge, 5% for the left multifidus, 20% for the right multifidus, and 30% for the left multifidus in the crawling position with raised right arm and raised left leg (Imai et al., 2010). The findings above indcate that the ergometer exercise as a core strengthening exercise is slightly inferior to the commonly practiced exercise. However, it might represent a new type of exercise for patients who are unable to assume the posture to perform the commonly practiced core strengthening exercise.

The trunk muscles are classified into a local system, consisting of muscles that originate or stop at the lumbar spine and provide stability of the spinal segments, and a global system, consisting of muscles that originate at the pelvis and stop at the thorax, which control movement of the entire spine and provide general trunk stability (Bergmark, 1989). Muscles in the local system are permanently activated at a low level, regardless of movement, whereas the global system is movement-dependent and activated. The superficial fibers of the multifidus show activity similar to that of the erector spinae during arm exercise in young, healthy participants, while the deep fibers of the multifidus always show low activity, indicating differences in the function of the superficial and deep fibers (Moseley et al., 2002). This study employed sEMG to measure muscle activity, and the multifidus measured were superficial fibers. Therefore, the increased load of the arm- and leg-ergometer exercise elicited increased activity in the erector spinae and multifidus, possibly because these muscles functioned as part of a global system. Marks et al. used sEMG to observe muscle activity in the erector spinae in healthy young men performing arm-ergometer exercise at 15W, 30W, and 45 W loads, and showed that muscle activity increased with increasing load (Marks et al., 2012). The loadings used in our study were higher than those used in that report, 50W and 100 W for both arm and leg extremities. The results suggest that higher loads are more useful for increasing paraspinal muscle activity.

Although there was no difference in the amount of increase in muscle activity in either the erector spinae or multifidus between arm- and leg-ergometer exercise, % MVC showed that arm-ergometer exercise stimulated more muscle activity in the erector spinae than leg-ergometer exercise. In a previous report, sEMG-measured activity of superficial fibers of the multifidus and thoracic erector spinae was larger during erect sitting than slump sitting, indicating that differences in posture affect muscle activity (O'Sullivan et al., 2002). In this study, the arm-ergometer exercise was performed during erect sitting, because the electrodes were placed on the back and the participant was instructed not to lean back and to sit shallowly against the seat surface. Alternatively, during the leg-ergometer exercise, the seat height was only set with the right knee joint at 0° extension, and all participants were in a posterior pelvic tilt. Therefore, differences in the pelvic anterior-posterior tilt angle during arm- and leg-ergometer exercise may have affected the results at rest and during exercise. However, there was no significant difference in the multifidus during arm and leg exercises, suggesting that the erector spinae and the multifidus may be differentially affected by posture during exercise. The results of the present study indicate that the arm ergometer was superior in eliciting activity in the erector spinae, while both arm and leg ergometers could stimulate the multifidus.

HR from the arm-ergometer exercise was higher than the leg-ergometer exercise at 100 W condition. Previous reports indicate that maximal oxygen uptake is 30%–50% larger during arm exercise than during leg exercise (Calbet et al., 2007; Helge, 2010) and that arm exercise at the same load is more likely to increase HR than leg exercise. Notably, arm extremities have less muscle capacity than the leg extremities (Calbet et al., 2005). Therefore, arm-ergometer exercise, which promotes higher HR, was considered superior in strengthening cardiopulmonary function.

### 5.1 Limitations

This study included healthy adult males and did not measure habitual physical activity levels. Previous studies have reported differences in skeletal muscle mass based on habitual physical activity level, sex, and age (Lexell et al., 1988; Janssen et al., 2000; Ramsey et al., 2021). It is well known that the number of motor units and the firing rate of motor units decrease with aging. These changes induce a decrease in EMG amplitude in the elderly (Roos et al., 1997). Therefore, we do not believe that the results of this study can be transferred to the general population with different sexes, ages, and habitual physical activities. Further studies are needed for those populations.

In this study, only the superficial fibers of the multifidus were evaluated because sEMG was used, and the deep fibers of the multifidus were not evaluated. Furthermore, only the erector spinae and the multifidus were the only muscles evaluated in this study. A previous study reported that the superficial and deep fibers of the multifidus function differently (Moseley et al., 2002), and that patients with fatty infiltration of the multifidus had less fatty infiltration of the psoas muscle as compensation (Özcan-Ekşi et al., 2021). Therefore, further studies are needed to include the deep fibers of the multifidus, as well as the psoas muscle.

The angle of anterior-posterior tilt of the pelvis in the sitting posture has been shown to affect the muscle activity of the erector spinae and multifidus (O'Sullivan et al., 2002). In this study, posture was defined by the angle of the joints of the upper and lower limbs. Therefore, future studies must define more detailed motor postures and evaluate them including the angle of anterior-posterior tilt of the pelvis.

The results of this study indicate that upper and lower extremity ergometer exercise activates the erector spinae and multifidus in a load-dependent manner. However, the effects of ergometer exercise over a period of time on the erector spinae and multifidus are not known. Therefore, the actual training effects on the erector spinae and multifidus should be verified in future studies.

## Conclusion

In the arm- and leg-ergometer exercises, muscle activity in the paraspinal muscles increased with increasing load. In the erector spinae, activity was higher in the arm, which may have been influenced by the angle of anterior-posterior pelvic tilt in the posture. The results of this study showed that both arm- and leg-ergometer exercises are potentially alternative methods of paraspinal muscle training for healthy participants. Further research is needed for the elderly and frail individuals who have difficulty implementing trunk training.

## Data Availability

The original contributions presented in the study are included in the article/supplementary material, further inquiries can be directed to the corresponding author.
